# Keeping track of time: Horizontal spatial biases for hours, days, and months

**DOI:** 10.3758/s13421-023-01508-1

**Published:** 2023-12-28

**Authors:** Anastasia Malyshevskaya, Alex Miklashevsky, Martin H. Fischer, Christoph Scheepers, Yury Shtyrov, Andriy Myachykov

**Affiliations:** 1 Potsdam Embodied Cognition Group, Cognitive Sciences, University of Potsdam, Karl-Liebknecht-Str. 24/25, D-14476 Potsdam-Golm, Germany; 2 Centre for Cognition and Decision Making, Institute for Cognitive Neuroscience, HSE University, Krivokolenniy Pereulok 3, Entrance 2, Moscow, Russian Federation 101000; 3 School of Neuroscience and Psychology, University of Glasgow, 62 Hillhead Street, Glasgow, G12 8QB UK; 4 Center of Functionally Integrative Neuroscience (CFIN), Institute for Clinical Medicine Aarhus University, Universitetsbyen 3, bldg 1719, 8000 Aarhus, Denmark; 5 Department of Psychology, Northumbria University, Northumberland Building, Newcastle upon Tyne, NE1 8ST UK

**Keywords:** Time words, Mental time line, Line bisection, Space-time association, Mouse tracking

## Abstract

In many Western cultures, the processing of temporal words related to the past and to the future is associated with left and right space, respectively – a phenomenon known as the horizontal Mental Time Line (MTL). While this mapping is apparently quite ubiquitous, its regularity and consistency across different types of temporal concepts remain to be determined. Moreover, it is unclear whether such spatial mappings are an essential and early constituent of concept activation. In the present study, we used words denoting time units at different scales (hours of the day, days of the week, months of the year) associated with either left space (e.g., *9 a.m.*, *Monday*, *February*) or right space (e.g., *8 p.m.*, *Saturday*, *November*) as cues in a line bisection task. Fifty-seven healthy adults listened to temporal words and then moved a mouse cursor to the perceived midpoint of a horizontally presented line. We measured movement trajectories, initial line intersection coordinates, and final bisection response coordinates. We found movement trajectory displacements for left- vs. right-biasing hour and day cues. Initial line intersections were biased specifically by month cues, while final bisection responses were biased specifically by hour cues. Our findings offer general support to the notion of horizontal space-time associations and suggest further investigation of the exact chronometry and strength of this association across individual time units.

## Introduction

One distinct feature of *Homo sapiens* is their ability to process complex abstract concepts (e.g., Borghi et al., 2018, 2022). Abstract concepts are those that have referents that cannot be perceived directly via sensory input (e.g., words like *creativity* or *legend*, but also words referring to emotions, time units, or quantities). Although they are omnipresent in human languages and are well described in the linguistic, psycholinguistic, and cognitive literature (see for reviews: Hoffman, 2016; Mkrtychian et al., 2019; Montefinese, 2019), the nature of abstract concepts is still a matter of debate. According to embodied theories of cognition, the sensorimotor system plays a key role in acquiring and retrieving not only concrete but also abstract concepts (Barsalou, 1999; Borghi et al., 2017; Myachykov et al., 2014; Pulvermüller, 1999; Vigliocco et al., 2004). For example, right-handers have been found to make faster responses to positive emotional words with their right hands, while in left-handers, this pattern is reversed (Casasanto, 2009), supporting the notion of spatial mapping of affective semantics. Another example is the influence of breathing rhythms on processing quantities, leading to the perception and production of larger numbers after inhaling compared to exhaling (Belli et al., 2021), in line with the framework of linking numerical cognition to the physical experience of magnitude (Sixtus et al., 2023).

The present study focuses on time concepts – a particularly frequent, practically relevant, yet challenging concept type. While highly abstract, they are among the first words that appear in the human lexicon, as well as among the most commonly used words in any language (Kopár, 2010). Although processing information about time might be crucial for survival in the dynamic world around us, people are not known to possess special receptors for time perception. This constitutes a challenge for the embodied cognition framework (but see Borghi et al., 2022, for counterarguments). Here, we investigate how activation of time concepts is reflected in putatively related sensorimotor processes.

Existing research suggests that people often rely on space when talking about time. For example, processing spatial stimuli can prime reasoning about past and future events (Boroditsky & Ramscar, 2002). Moreover, time-related phrases often include space-related words, such as *an hour behind* or *the days ahead* (see Gentner et al., 2002; Lakoff, 1993). Time-related expressions are also often accompanied by gestures systematically pointing to specific directions (e.g., Núñez et al., 2012; Núñez & Sweetser, 2006; Walker & Cooperrider, 2016).

One well-documented demonstration of the association between time and space is the phenomenon as known as Spatial-Temporal Associations of Response Codes (STEARC): facilitated left-oriented responses to past-related words and right-oriented responses to future-related words (e.g., Ishihara et al., 2008; Santiago et al., 2007; Torralbo et al., 2006). Initially observed in the horizontal dimension, the STEARC effect was later registered in the vertical dimension as well, with past-related words associated with lower space and future-related words with upper space (for European cultures: e.g., Beracci & Fabbri, 2022; Ruiz Fernández et al., 2014; but see Boroditsky et al., 2011; Casasanto & Bottini, 2014, Experiment 1; Kolesari & Carlson, 2018, for the absence of vertical STEARC effect for certain types of temporal stimuli). Finally, a similar space-time association was found for the sagittal dimension, with past events occupying the space behind and future events in front of the agent (e.g., Teghil et al., 2021). As an important point to note, however, linear mappings are not the only spatial arrangements available for the mapping of temporal concepts. Several studies indicate a circular clockwise representation for months of the year, with January positioned either at the top or at the bottom of an imagined circle (Laeng & Hofseth, 2019; Leone et al., 2018; see also Seymour, 1980). A similarly circular format has been inferred for hours of the day (Bächtold et al., 1998; Ristic et al., 2006; Vuilleumier et al., 2004), probably reflecting time representation on a standard clock face.

Taken together, these and similar findings suggest that time concept representations can be organized within a complex three-dimensional space that includes horizontal, vertical, and sagittal axes (Boroditsky, 2011; Ding et al., 2020; Miles et al., 2011). Among the three axes, or *Mental Time Lines* (MTL, Bender & Beller, 2014; Bonato et al., 2012), the horizontal dimension has received particular attention in studies using reaction-time analysis (see von Sobbe et al., 2019, for a meta-analysis), although there is also considerable research on the sagittal dimension, such as that on the “Wednesday’s meeting task” (Bender et al., 2010; Matlock et al., 2005) and the temporal focus hypothesis (Bylund et al., 2020; Callizo-Romero et al., 2020; de la Fuente et al., 2014).

The direction of the horizontal MTL is most likely influenced by reading and writing habits or conventions (see Bender & Beller, 2014, for review; see also Boroditsky et al., 2011; Chen & O'Seaghdha, 2013). Indeed, the MTL is oriented from left to right in cultures using left-to-right reading and writing systems (Bergen & Lau, 2012; Ouellet, Santiago, Funes, & Lupiáñez, 2010b), while the opposite direction is commonly found in cultures with right-to-left reading systems (for Hebrew native speakers see, e.g., Fuhrman & Boroditsky, 2010; Ouellet, Santiago, Israeli, & Gabay, 2010a). At the same time, more recent research has considered additional influences on the horizontal MTL direction, e.g., calendars, graphs, and individual experience (Pitt & Casasanto, 2020; Starr & Srinivasen, 2021).

Regardless of the specific axes used in these individual studies, the resulting theoretical accounts aiming to explain the general principles of spatial-temporal associations tend to be *universalist* – i.e., they try to explain how time is conceptualized and represented *in general,* regardless of the specific linguistic categories, word classes, or time units (e.g., Walsh, 2015). There are a number of theories that contribute to such a universalist approach, including A Theory of Magnitude (Walsh, 2003) and Conceptual Metaphor Theory (Lakoff, 1993). For example, Walsh (2003) argues that the inferior parietal cortex serves as a universal hub supporting spatial-conceptual mappings across distinct conceptual domains including time and number concepts. In a more general sense, this approach assumes both that the representations belonging to *different* domains (e.g., time and number) and different categories within an individual domain (e.g., hours, days, and months) should have a common neurocognitive source and, as a result, manifest similar spatial-conceptual mapping signatures in behavioural studies. Accordingly, the same representations are presumed to underlie all temporal concepts, and therefore these concepts should in principle be mapped uniformly. This universalist conceptualization of the MTL is motivated by findings from a diverse range of time-related phenomena, such as time-related words and expressions (e.g., Beracci et al., 2022; Santiago et al., 2007; Torralbo et al., 2006), visually presented past and future events (Fuhrman & Boroditsky, 2010; Santiago et al., 2010), and individual time-unit types, for example, months, days, and hours (see below). Equally diverse are the experimental paradigms used in these and other studies. Put together, while existing evidence provides strong support for regular time-space associations, there has been little effort thus far to investigate the regularity and consistency of *the same* time-space associations (1) across different types of temporal concepts and (2) using the same experimental paradigm. In an ideal scenario, one would want to use all three mapping axes (horizontal, vertical, and sagittal) combined with the same experimental task in order to attest to the uniform arrangement of time concepts in a three-dimensional space. However, this may be challenging from a practical point of view because, in paradigms relying on 2D (e.g., computer screen) presentation, there are natural limits on the degree to which the sagittal space can be represented. Moreover, given that findings from studies using the vertical dimension are quite inconsistent (as well as the theoretical accounts of *why* time should flow from bottom to top or the other way around; for a review, see Dalmaso et al., 2023), the horizontal dimension is most suitable for a comparison of spatial biases exerted by different time concepts. Thus, the present study aims to achieve exactly that – to examine, using the same experimental paradigm, the horizontal space-time mapping for three distinct groups of time words that operate on different timescales – namely, hours, days, and months.

Gevers et al. (2003) were the first to report the horizontal STEARC effect for months. Their study demonstrated that, for native speakers of Dutch, months are arranged from left to right, starting in January and ending in December. Gevers et al. (2004) later found the same horizontal arrangement for days of the week, with Monday on the left and Friday on the right. While there is scarce research following the same approach in order to analyze the spatial-conceptual mapping of the hours of the day, this expectation naturally follows from universalist approaches to space-time associations described above. This prediction also follows, for example, from a study by Ding et al. (2015) demonstrating that the conventional periods within the day (i.e., morning, afternoon, and evening) also exhibit a left-to-right mapping.

As mentioned above, most studies tend to investigate the horizontal mapping of distinct time units separately (Di Bono & Zorzi, 2013; Franklin et al., 2009; Gevers et al., 2003; He et al., 2020; Zorzi et al., 2006: months; Dodd et al., 2008: months and days; Leone et al., 2018: months, days, and parts of the day; Gevers et al., 2004: days). One recent exception is a study by Malyshevskaya et al. (2022), who used the same experimental protocol to examine horizontal spatial biases on three time units of different scales. In that study, participants indicated by a mouse click the locations of individual time units (hours, days, and months; e.g., *9 a.m., Friday, November*) on visually presented line segments representing different time periods. Therefore, each word corresponded to the left, right, or central position on a line in different conditions. Analysis of (1) manual response latencies and (2) their horizontal coordinates in congruent vs. incongruent conditions (defined via the combination of word meaning and the position of the line) demonstrated a general horizontal mapping of all time units, but the strength of this association varied across dependent measures as a function of time unit type. More specifically, a reaction time facilitation effect was revealed for hours and days but not for months. Moreover, a rather complex pattern was observed in the manual response coordinates; for example, while a congruency effect was registered in right-biasing months (e.g., *November*), the same was not true for left-biasing months (e.g., *March*).

We hypothesize that this partial inconsistency can be attributed to several factors. One of these factors is the potential impact of the visual cueing manipulation used in that study (the stimulus location on a line), which may have resulted in the participants’ bottom-up perceptual biases masking or contradicting the presumed top-down conceptually driven ones. More importantly, the authors focused on somewhat delayed response-related measures – overt reaction times and final response coordinates (see, e.g., von Sobbe et al., 2019, for a meta-analysis of studies using reaction time). It therefore remains unclear whether the spatial signatures of MTL activation accrued during the processing of time-related words are present at the earlier stages of semantic processing or instead reflect secondary, post-comprehension effects associated with response planning. This clarification is important because it adjudicates competing theoretical views about the human mind. Theories of embodied cognition argue that sensory and motor activations accompany word access at the earliest stages of conceptual processing and form an obligatory constitutive part of concept activation (e.g., Hauk et al., 2004; Lynott et al., 2020), not merely a late-emerging epiphenomenon, as proposed by more traditional representational theories of conceptual knowledge (see reviews in Fischer & Zwaan, 2008; Meteyard et al., 2012).

## Current study

In the present study, we combined a classical version of the line bisection task with the mouse tracking technique (see below for more details) aiming to (1) verify previous results of diverging horizontal spatial biases for time units of different scales (Malyshevskaya et al., 2022, reviewed above) without potential bottom-up confounds and (2) extend these findings by additionally investigating response parameters that are known to reflect early and implicit processes of spatial-conceptual mapping, previously unexplored in this type of experiments, namely (i) continuous tracking of the mouse cursor trajectory and (ii) the coordinates of the initial intersection between the cursor and the line – both well preceding the overt bisection response. This novel approach allowed us to assess sensorimotor activation reflected in both overt and covert processes that may accompany spatial-conceptual mappings of time units in horizontal space.

The present study had two general theoretical goals. First, we aimed to examine whether processing of distinct time units – hours of the day, days of the week, and months of the year – invokes spatial biases that are reflected in both the continuous engagement of the motor system (via mouse tracking) and perceptual judgments of midpoints of horizontal lines (via performance in a line bisection task). Especially the former measure can tell us whether space-time association is an early and constitutive part of time concept activation. Second, we aimed to assess the regularity and consistency of horizontal time-space associations across the aforementioned time units. Specifically, we intended to elaborate on previous findings and to test the uniformness of horizontal spatial-temporal mapping for different types of temporal concepts.

For these purposes, we compared mappings of distinct time units along the horizontal dimension using the same experimental task: *line bisection* (for review, see Jewell & McCourt, 2000). Line bisection task allows for assessing visuospatial biases and attentional shifts while performing simple perceptual judgments. In the original version of this task, participants indicate the midpoint of a straight horizontal line. Although healthy participants show only a small leftward deviation in line bisection, semantic processing is known to induce further shifts/biases in their accuracy (Fischer, 2001a). The line bisection task has been widely used to investigate associations between space and emotion (Cattaneo et al., 2014; Milhau et al., 2017; Tamagni et al., 2009), numbers (Fabbri & Guarini, 2016; Fischer, 2001b), and musical tones (Hartmann, 2017; Ishihara et al., 2013; Lega et al., 2014). For example, a study by Milhau et al. (2017) showed that the processing of positively valenced words was accompanied by rightward/leftward bisection biases in right-/left-handed participants, respectively. Thus, the line bisection task can potentially engage motor, attentional, and other cognitive processes (cf., Fischer, 2001a). However, to our knowledge, this potential has only very scarcely been used in MTL research on healthy individuals. Filling this gap was therefore an additional aim of the present study.

Regarding the issue of early and constitutive versus late-emerging sensorimotor activation, we address this debate by examining the behavioral impact of different time units with a highly time-sensitive method: *mouse tracking*. Tracking mouse movements during experimental task performance allows us to access unfolding cognitive processes in their temporal and spatial dynamics (Spivey & Dale, 2006). Mouse tracking has been used to investigate the processing of action words (e.g., Kamide et al., 2016), cardinal directions (e.g., Tower-Richardi et al., 2012), emotion (e.g., Mattek et al., 2016), number (see Faulkenberry et al., 2018, for review), and other magnitude-related stimuli (e.g., pitch of musical tones; Hartmann, 2017). However, we are only aware of one study using mouse tracking to investigate space-time associations. Miles et al. (2010) demonstrated sensorimotor activation already 600 ms before the actual line bisection response was provided: Participants’ mouse-movement trajectories deviated more to the left while processing past-related information and more to the right while processing future-related information.

Applied to the line bisection task, mouse tracking allows for the registration of sensorimotor activation even when more explicit bisection response biases remain absent (Hartmann, 2017; Haslbeck et al., 2016) because the *approach trajectories* of the mouse cursor towards the line contain real-time evidence about ongoing sensory-motor control processes in the brain. For example, Hartmann (2017) applied mouse tracking in the line bisection task to investigate whether musical tones induce shifts of spatial attention. In his experiment, participants bisected a horizontal line while listening to low- versus high-pitched tones. Hartmann analyzed not only the *eventual line bisection* coordinates but also the *initial line intersection* coordinates (when participants approached the line for the first time but had not yet made any corrections) as well as the trajectories of cursor movement towards the line. This allowed for the investigation of late, intermediate, and early emerging spatial biases, respectively. The results showed that, although processing musical tones did not influence final bisection responses, it nevertheless affected the initial line intersection coordinates and movement trajectories. Thus, mouse tracking represents a powerful instrument to investigate subtle spatial biases at very early stages of sensorimotor activation during time unit processing.

The line bisection task requires *implicit* activation of spatial and temporal representations. In a recent meta-analysis, von Sobbe et al. (2019) suggested that the activation of the MTL depends on the extent to which time is relevant to the experimental task. Indeed, most studies reporting strong space-time associations employed tasks that required categorizing temporal references of the stimuli (e.g., Ding et al., 2015; Eikmeier et al., 2015; Santiago et al., 2007). At the same time, studies in which time was irrelevant to the task showed weaker or no such effects (e.g., Dalmaso et al., 2023; Flumini & Santiago, 2013; Maienborn et al., 2015; Ulrich & Maienborn, 2010). However, some notable recent exceptions show sensorimotor effects in implicit tasks (e.g., Grasso et al., 2022; Topić et al., 2022), thereby suggesting that space-time association might nonetheless be an early and automatic process. In the present study, we asked participants to perform simple perceptual judgments while listening to temporal words, thus avoiding explicit categorization of temporal references. At the same time, we ensured continuous lexical access to those time words by administering verification questions (see details of the procedure below).

To sum up, we adopted a line bisection task combined with auditory semantic stimulation and mouse tracking, as in Hartmann’s (2017) study reviewed above. We expected to register a specific pattern of sensorimotor activation while processing time units. Specifically, past-associated temporal words should lead to left-oriented, and future-associated temporal words to right-oriented attentional shifts, similar to the effects observed in Posner’s classical attentional cueing paradigm with spatially meaningful symbols (Posner, 1980; for a recent review, see Shaki & Fischer, 2023). Posner (1980) demonstrated that the processing of a centrally presented arrow pointing to the left or to the right resulted in the deployment of participants’ attention toward the cued location and facilitated target-detection responses. Moreover, we expected sensorimotor activation to be detectable at the early stages of cognitive processing and thus to be reflected in corresponding mouse-trajectory shifts during line approach of the mouse cursor and in initial line intersection coordinates. Finally, based on our previous research (Malyshevskaya et al., 2022), we expected horizontal sensorimotor mappings to be registered for all three time units (hours, days, months) but with potential differences in their association strength. Although all three time unit types have previously shown associations with the horizontal MTL (Ding et al., 2015; Gevers et al., 2003; Gevers et al., 2004), we might expect that horizontal associations are stronger for hours of the day than for days of the week and months of the year.

### Participants

In order to estimate the sample size necessary to register a moderate effect, we referred to a meta-analysis by von Sobbe et al. (2019), who reported an average effect size of *d* = 0.46. This paper concluded that “for a statistical power of 0.90, at least 41 participants are needed when manipulating the space-time congruency effect within subjects” (von Sobbe et al., 2019, p. 16). A similar number of participants (40.69 on average) was specified in the power estimation reported by Beracci and Fabbri (2022). To further increase the likelihood of detecting a medium effect and protect against data loss from drop-outs or poor performance, we increased our sample size to 61. Four participants’ data sets were excluded from analysis as they did not understand the instruction correctly: More than 25% of their responses were distributed along the entire line and not within its middle part (±100 pixels; see *Procedure* for details below). As a result, 57 participants remained in the final sample (42 females, mean age 23 ± 5.9 years). All participants were healthy native Russian speakers, had normal or corrected-to-normal vision, and had no prior knowledge of the study design or hypotheses. The study was designed and conducted following the guidelines of the Declaration of Helsinki. All participants gave their informed consent before the beginning of the experiment. Participants were recruited using social networks; they were remunerated for their time and debriefed at the end of the session.

### Experimental design and materials

To investigate how individual time units are mapped onto horizontal space and whether this mapping is regular and consistent, we used Russian phrases denoting hours of the day (e.g., *семь часов утра - seven a.m.,* or literally ‘seven o’clock in the morning’) as well as words denoting days of the week (e.g., *воскресенье* – *Sunday*), and months of the year (e.g., *март* – *March*). Similar to many other European languages, Russian is a language with a left-to-right reading and writing system and 24-h as well as 12-h time-reading formats, both widely used. The week in Russian begins on Monday and ends on Sunday and the year starts in January and ends in December. Equal numbers of left- and right-biasing stimuli were selected for each time unit type, resulting in a total of 18 stimulus items. Specifically, we selected three putatively left-biasing (*five a.m., seven a.m., nine a.m.*) and three right-biasing (*four p.m., six p.m., eight p.m.*) hour stimuli. Similarly, the stimulus set included three left-biasing (*Monday, Tuesday, Wednesday*) and three right-biasing (*Friday, Saturday, and Sunday*) names of the days of the week. Finally, we included three left-biasing (*February, March, May*) and three right-biasing (*August, September, October*) names for the months. As a result, the following two factors were independently manipulated in a 3 × 2 within-participants design: Time Unit (Hours/Days/Months) and Word Bias (Left/Right).

**Fig. 1:**
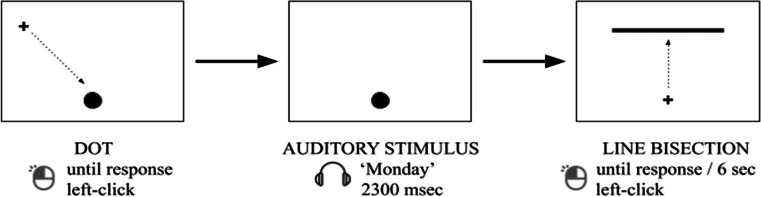
Example of stimuli and the experimental trial sequence (not to scale)

The mouse cursor had a black crosshair shape of a 30 × 30 px size. Time words were recorded (32-bit, sampled at 22,050 Hz) using a male synthesized voice in Yandex SpeechKit software (http://5btc.ru/voice/). Each auditory item was paired with two visual stimuli consisting of a black dot and a horizontal line (see Fig. 1). The black dot indicated the point from which the cursor movement was to be initiated (see *Procedure* for details). The dot was always of the same size (70 × 70 px). It was always presented at the bottom of the screen (450 px below the screen center), while its horizontal location varied (0-px, ±200-px, and ±450-px deviation from the center of the screen). The horizontal line represented the target stimulus. As per Hartmann’s (2017) protocol, this horizontal line varied in length (650 px, 750 px, and 850 px) and horizontal location (0-px, ±200-px, and ±450-px deviation from the center of the screen) across trials, while its vertical location remained constant (300 px above the center of the screen). Note that the horizontal location of the center of the line always corresponded to the horizontal location of the dot center. Overall, 15 horizontal lines were combined with the 18 time words listed above, thus resulting in a total of 270 unique trials.

### Procedure

The experiment was implemented in PsychoPy © Version 3.2.3. The monitor specifications were 16:9, diagonal 21.5-in., and screen resolution 1,920 × 1,080 pixels. Participants were seated in front of the screen at an approximate visual distance of 60 cm. Each participant was tested individually in a soundproof booth. The experiment began with a short practice session consisting of nine trials. During this training session, participants were presented with stimuli not used in the main experiment: hour units (*11 a.m., 7 p.m., 9 p.m.*), day units (*Thursday* (repeated thrice)), and month units (*April, January, December*). The main experimental session consisted of two blocks with a short break in-between; each block consisted of 270 individually randomized trials. Each trial started with a dot presented at the bottom of the screen. Simultaneously with the onset of a dot, a mouse cursor (cross) appeared at a random location on the screen. Participants used the mouse to click on the center of the dot. This triggered the onset of the auditory stimulus presentation via headphones (Fig. 1, leftmost panel). During the auditory stimulus presentation, it was impossible to move the mouse cursor; it remained in the center of the dot during the entire duration of the audio (Fig. 1, middle panel). Upon the offset of the auditory stimulus, the dot disappeared (leaving the mouse cursor), and a horizontal line was presented at the top of the screen (Fig. 1, rightmost panel). Participants were instructed to indicate (via point-and-click) the apparent center of the line as quickly and accurately as possible. Note that participants always started the cursor movement from the location where the center of the dot had been presented; they could only start moving the cursor after the auditory stimulus ended. Thus, movement onsets on average corresponded to the following starting times: 1,241 ms for hours, 888 ms for days, and 686 ms for months after the auditory stimulus onset. The horizontal line remained on the screen until a response was recorded, with a timeout of six seconds. To ensure cognitive processing of the linguistic cues, participants answered verification questions about the last presented word in 40% of trials. These verification questions were about the relevant time unit of the word (e.g., “Did the word you just heard refer to a month of the year?”) but, importantly, *not* about its location on the mental time line (past, present, future). Participants answered by pressing the M key for “yes” and the C key for “no.” A random half of the verification questions required “yes” and the other half “no” as the correct answer.

## Data preprocessing and analysis

Following the protocol used by Hartmann (2017), we computed three dependent measures: (1) *movement trajectory* (x and y coordinates of cursor positions within the determined time windows from the line onset to the participant’s final response); (2) *initial line intersection coordinates* (x-coordinates of the points at which participants first crossed the line before adjusting their bisection responses); and (3) *final bisection response coordinates* (x-coordinates of participants’ line-bisection responses).

For the movement trajectory analysis, the *mousetrap* package (Wulff et al., 2021) in R version 4.1.3 (R Core Team, 2020) was used. The starting points of all trajectories were aligned, and cursor coordinates were time-normalized to 100 data points, with each data point being about 12-ms duration on average. For data trimming, responses with a maximum absolute deviation from the straight line (a direct path connecting start and end trajectory points) of more than 2.5 standard deviations from the mean (by participant and by condition) were excluded from the analysis. This left us with 97.7% of the data, which were subjected to further statistical analyses. Finally, movement trajectories of left and right Word Bias conditions were calculated and tested against each other for each data point using Wilcoxon Signed-Ranks statistics (we chose a nonparametric test since the error distribution clearly deviated from a standard Gaussian). Following Hartmann (2017), we considered differences significant if *p*-values were below .05 for at least five consecutive data points.

For the initial line intersection analysis, we excluded trials with a maximum horizontal deviation of more than 150 px from the line center. The remaining 91.4% of the data were subjected to statistical analyses. To accommodate the residual continuous cursor movement during the final bisection response, the coordinate deviation filter was narrower, including values with a maximum horizontal deviation of no more than 100 px from the line center. The statistical analysis was performed on the remaining 99.6% of these data. Both types of response coordinate data were analyzed using within-participant ANOVA models with Time Unit (Hours/Days/Months) and Word Bias (Left/Right) as factors, followed by post hoc pairwise t-tests.

## Results

### Movement trajectory

**Fig. 2:**
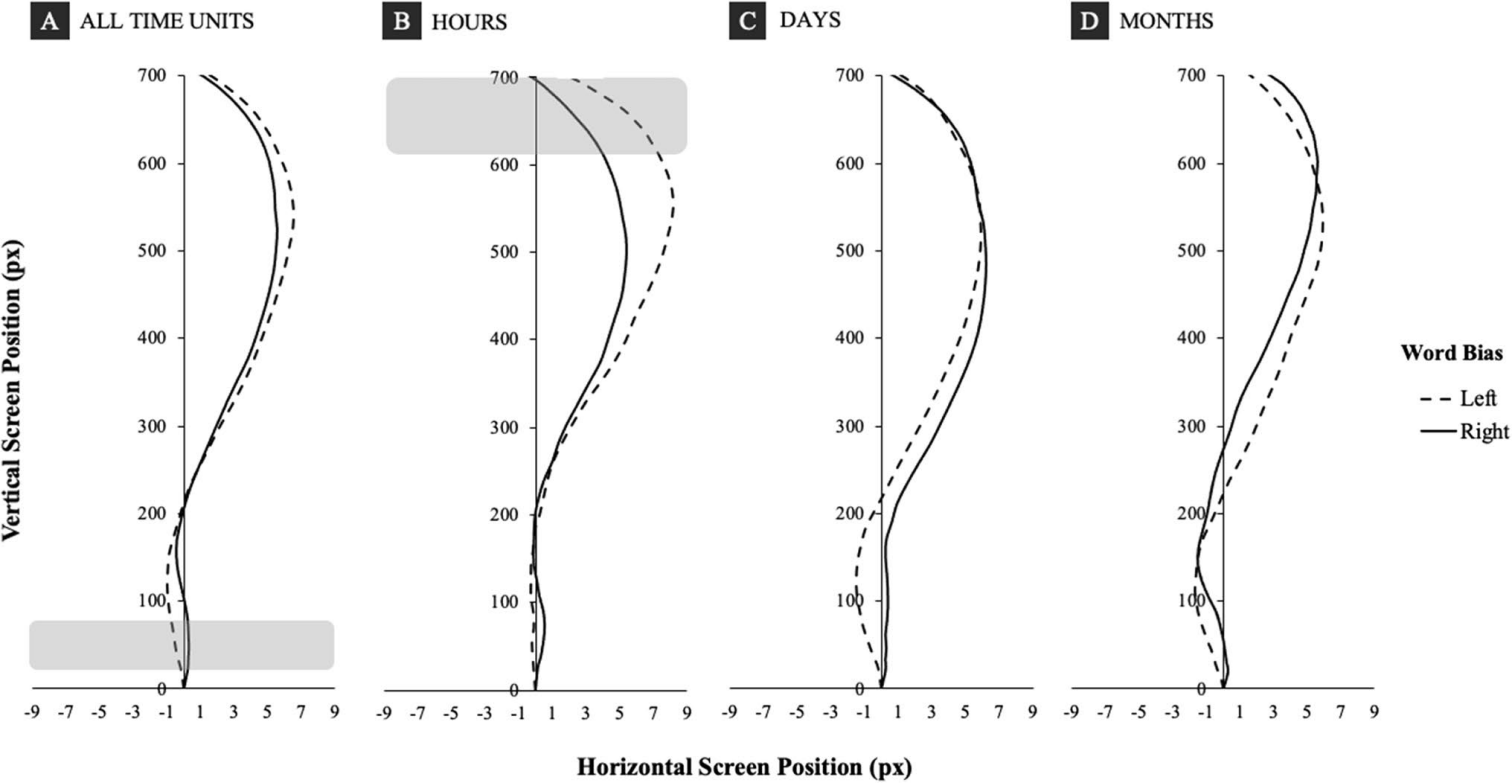
Movement trajectories (time-normalized data). Left versus right word biases for (**A**) all time units, (**B**) hour units, (**C**) day units, and (**D**) month units. Grey areas represent the periods of statistically significant differences between conditions (Wilcoxon Signed-Ranks tests) over at least five consecutive data points

We analyzed movement trajectory data to examine whether the processing of time units leads to spatial biases reflected in a continuous hand movement. Results are depicted graphically in Fig. 2;^1^ full statistical results are also available in an online repository (see *Data Availability Statement* below for the link). The analysis revealed a general pattern with a slight initial leftward drift (around the first 20% of the movement) followed by a strong rightward drift (in the remaining part of the movement, see Fig. 2A, B, and D). Within this general pattern, reliable differences between left- and right-biasing time units were found in the first five data points (i.e., approximately 0–60 ms after the movement onset): Mouse trajectories deviated more to the left when participants processed left-biasing words and more to the right when participants processed right-biasing words (see Fig. 2A). At the same time, between the 30th and the 41st data points (i.e., around 360–490 ms after the movement onset), trajectories for left-biasing hour units were significantly more rightward when compared to the right-biasing time units (see Fig. 2B).

**Fig. 3:**
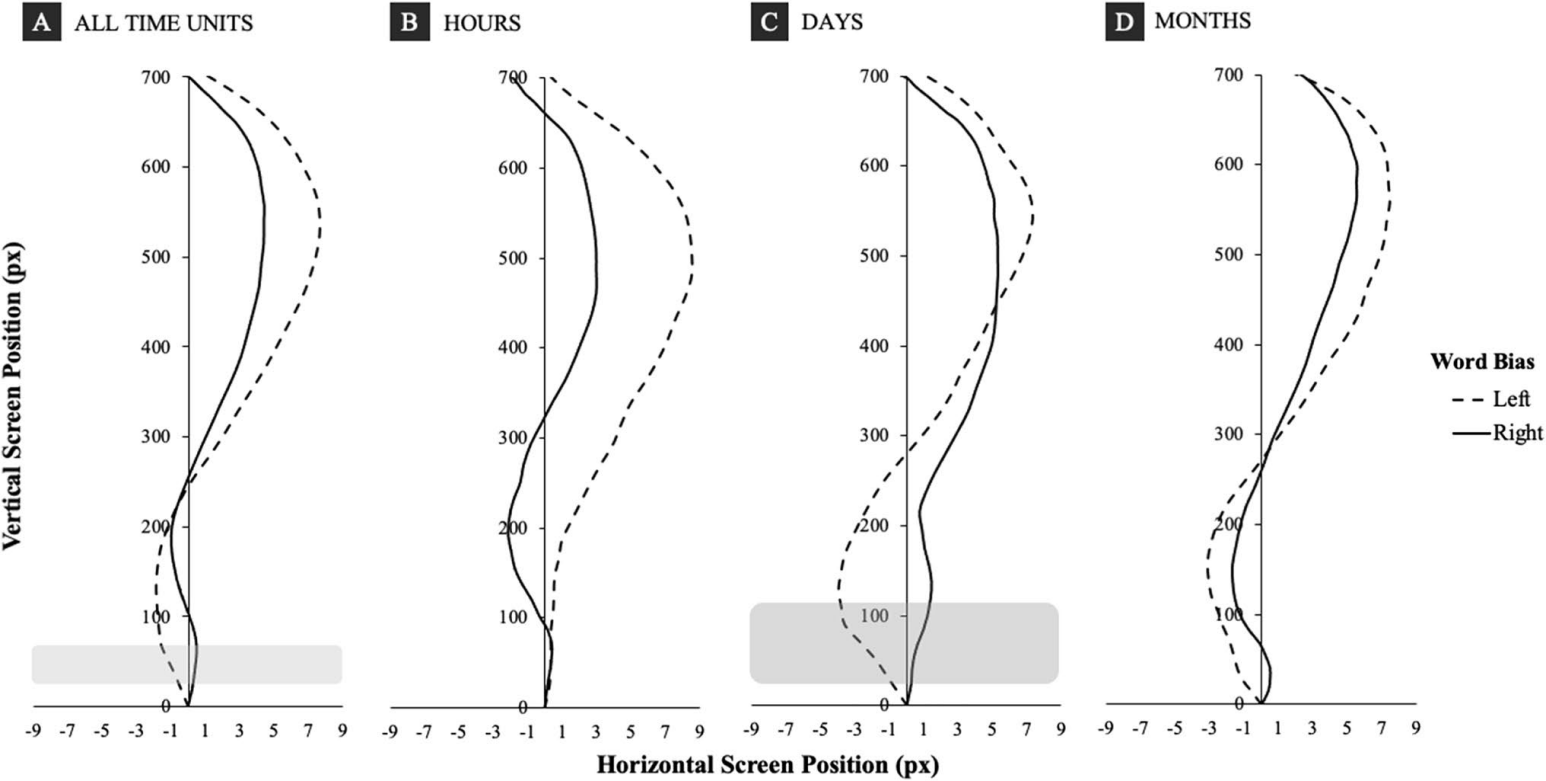
Movement trajectories for extreme points (time-normalized data). *Extreme* left versus right word biases for (**A**) all time units, (**B**) hour units, (**C**) day units, and (**D**) month units. The dark-grey area represents the period of statistically significant (Wilcoxon Signed-Ranks tests) differences between conditions over at least five consecutive data points, the light-grey area – over four consecutive data points

We also contrasted movement trajectories for *extreme time units* only (in the present set, *February / November, Monday / Sunday, 5 a.m. / 8 p.m.*). For this purpose, the same data preprocessing and analysis steps were performed as described in the main analysis above. The results are shown in Fig. 3, and a table with full statistical results is also available online (see *Data Availability Statement*). We found reliable differences between left- and right-biasing time units overall (Fig. 3A), but only in the first four data points (i.e., approximately 0–50 ms from stimulus onset). Furthermore, we found significant differences in the first six data points (i.e., approximately 0–70 ms from stimulus onset) for days of the week, with trajectories deviating more to the left for left-biasing days and more to the right for right-biasing days (Fig. 3C).

### Initial line intersection coordinates

**Fig. 4:**
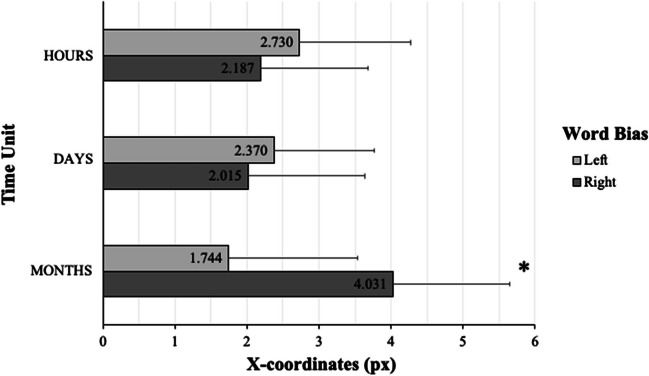
Initial line-intersection coordinates, group-mean data. Initial line intersection biases (in pixels) as a function of (left vs. right) Word Bias and Time Unit (hours, days, months). Error bars represent standard errors for the means. Zero represents the true line midpoint, and more positive values indicate more rightward deviations from the true line midpoint

Initial line intersection coordinates were analyzed to examine whether processing of time units leads to spatial biases in continuous hand movements before participants provide final perceptual judgments. The statistical results are presented in Table 1. The analysis showed that, while there were no reliable main effects of either Time Unit (*F*(2,112) = .368, *p* = .693) or Word Bias (*F*(1,56) = .808, *p* = .373), there was a trend for the interaction between these factors (*F*(2,112) = 3.080, *p* = .050). While this interaction’s significance is exactly at the conventional threshold, our specific prior hypotheses regarding differences between individual conditions motivated the use of post hoc comparisons (e.g., Wei et al., 2012). Examination of the interaction using pairwise t-tests revealed a significant difference between left- and right-biasing month units (*t*(56) = -2.415, *p* = .019) with a shift in participants' responses more to the right for right-biasing month units (*Mean* = 4.0, *SD* = 12.2) in comparison with left-biasing month units (*Mean* = 1.7, *SD* = 13.5) (see Table 2; Fig. 4).

**Table 1:** ANOVA: Interaction between Time Unit and Word Bias (X-coordinates, initial line-intersection)

Variance	*Df*	*F*	*p*	*η*_*p*_^*2*^
Time Unit	2, 112	.368	.693	.007
Word Bias	1, 56	.808	.373	.014
Interaction Time Unit * Word Bias	2, 112	3.080	.050	.052

**Table 2:** Pairwise t-tests: Interaction between Time Unit and Word Bias (X-coordinates, initial line-intersection)

Time Unit	Word Bias	Effect	*SD*	*t*	*df*	*p*
Hours	left vs. right	.543	6.524	.629	56	.532
Days	left vs. right	.355	6.659	.403	56	.689
Months	left vs. right	-2.287	7.150	-2.415	56	.019

### Final bisection response coordinates

**Fig. 5:**
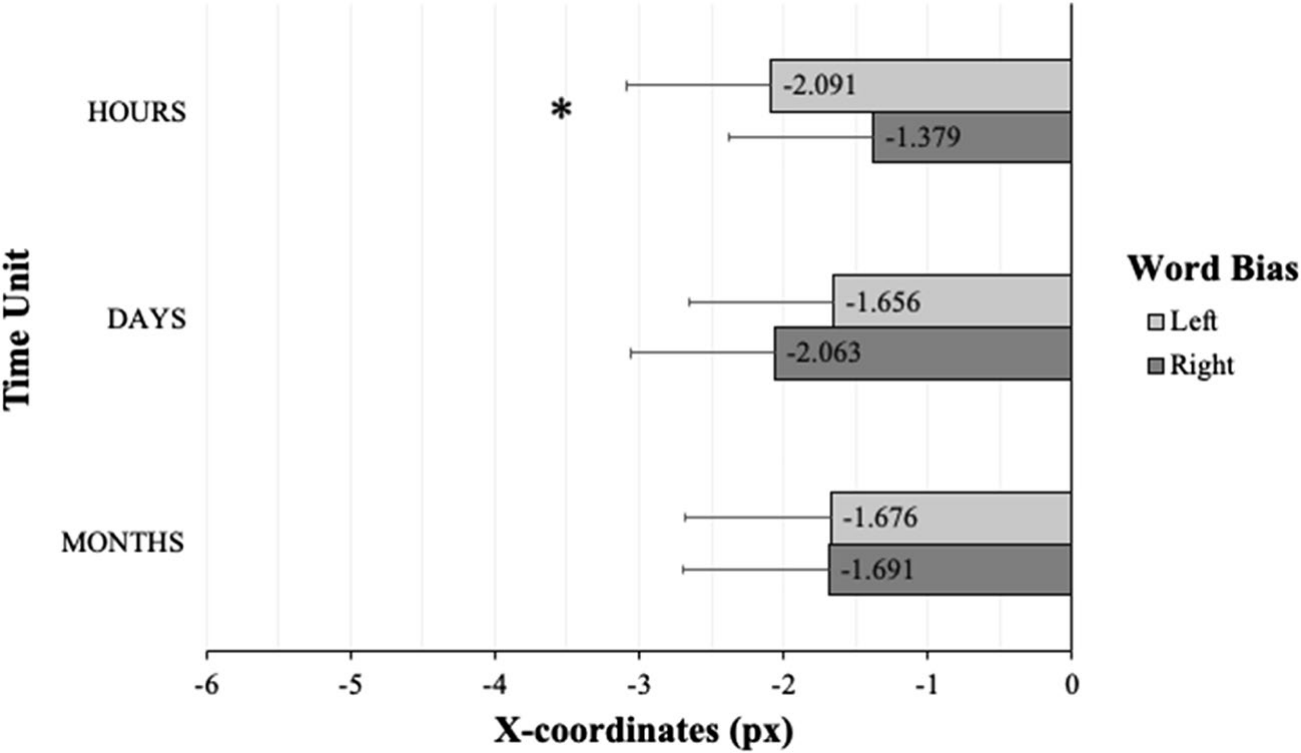
X-coordinates of the final bisection response: group-mean data. Final line bisection response biases (in pixels) as a function of (left vs. right) Word Bias and Time Unit (hours, days, months). Error bars represent standard errors of the means. Zero represents the true line midpoint; negative values indicate leftward deviations from the true line midpoint

We analyzed final bisection response coordinates to examine whether the processing of time units influences spatial biases as reflected in perceptual judgments of the midpoint of the presented horizontal line. The statistical results are presented in Table 3. Again, whereas the two main effects were not significant per se (Time Unit: *F*(2,112) = .274, *p* = .761; Word Bias: *F*(1,56) = .208, *p* = .650), we detected a statistical for the two-way Time Unit × Word Bias interaction (*F*(2,112) = 2.450, *p* = .091). As per the rationale described above, we performed pairwise comparisons even in the absence of a fully significant interaction (Wilcox, 1987) because we had specific prior hypotheses regarding differences between individual conditions (e.g., Wei et al., 2012). This analysis showed a significant difference between left- and right-biasing hour units (*t*(56) = -2.049, *p* = .045): Participants exhibited a stronger leftward bias after processing left-biasing hour units (*Mean* = -2.1, *SD* = 9.2) in comparison to right-biasing hour units (*Mean* = -1.4, *SD* = 9.0) (see Table 4; Fig. 5). Note that final bisection responses were generally biased to the left of the actual line bisection point. This general leftward bias is typical for readers of left-to-right reading languages and has already been documented in earlier line bisection studies (Chokron et al., 1998; Jewell & McCourt, 2000).

**Table 3:** ANOVA: Interaction between Time Unit and Word Bias (X-coordinates, final bisection response

Variance	*df*	*F*	*p*	*η*_*p*_^*2*^
Time Unit	2, 112	.274	.761	.005
Word Bias	1, 56	.208	.650	.004
Interaction Time Unit * Word Bias	2, 112	2.450	.091	.042

**Table 4:** Pairwise t-tests: Interaction between Time Unit and Word Bias (X-coordinates, final bisection response)

Time Unit	Word Bias	Effect	*SD*	*t*	*df*	*p*
Hours	left vs. right	-.712	2.623	-2.049	56	.045
Days	left vs. right	.406	2.931	1.047	56	.300
Months	left vs. right	.014	2.697	.040	56	.968

## Discussion

This study aimed at (1) investigating whether sensorimotor activation is reflected both in early cognitive processes and in final behavioral responses, and (2) examining the generality of the horizontal spatial-temporal mapping pattern. For this purpose, we compared mappings of distinct time units (hours of the day, days of the week, and months of the year) along the horizontal dimension, using mouse tracking in the line bisection task (Hartmann, 2017). Participants listened to temporal words with associated left versus right spatial biases and then bisected horizontal lines that varied in length and spatial locations. We investigated shifts in visual attention caused by processing these temporal word cues, registered as differences in *x* and *y* coordinates of the cursor movement trajectories, the initial line-intersection, and the final bisection response.

The first goal of our study was to examine whether sensorimotor activation emerges during the early stages of sensorimotor response following the apprehension of the word itself, as suggested by theories of embodied cognition. It is important to note that any cognitive event that includes a behavioral response is a multi-componential process in that it starts with the activation of a distributed mental representation underlying conceptual access and ends with an overt response, in our case, the physical mouse click on a visually presented line interval (cf., Posner, 1978). As a result, any cognitive process will include both covert and implicit as well overt and explicit subcomponents. While endpoint signatures of conceptual access have been investigated relatively extensively in cognitive research, less is known about the processes preceding the overt response. In our study, the continuous analysis of cursor trajectories and the analysis of the initial intersection coordinates has been an important attempt to examine the dynamic nature of embodied conceptual access, including processes under less direct conscious control (Spivey & Dale, 2006). Our findings show that MTL activation is detectable *early* in continuous hand movements (both mouse trajectories and initial line intersections) before the final intersection judgment, supporting the embodied view of language processing (Fischer & Zwaan, 2008). Interestingly, the general sensorimotor effect was significant during the first 50–70 ms of the cursor movement. Since our participants were allowed to move the mouse cursor only after stimulus offset, these effects might reflect the “tail end” of semantic processing taking place already prior to the movement onset. Thus, spatial biases in mouse trajectories were revealed at approximately 950 ms for day units and at approximately 1,600 ms for hour unit processing. These results are consistent with previous studies on number concepts, which detected sensorimotor activation after number processing in a period from approximately 1,000 to 3,000 ms (e.g., Myachykov et al., 2015, 2016). Our findings add to previous MTL research using evidence from movement trajectories (Miles et al., 2010), suggesting that sensorimotor activation may be detectable at both early and late stages of cognitive processing, depending on specific task conditions.

The second goal was to assess the regularity and consistency of horizontal time-space associations across different time units. The analysis of mouse movement trajectories showed an initial leftward drift for left-biasing temporal words and a rightward drift for right-biasing temporal words when averaging across time unit types. This finding provides evidence for a *general* automatic horizontal time-space association. Therefore, it is consistent with existing research showing general rightward-oriented MTLs in cultures with left-to-right reading directions (e.g., Bergen & Lau, 2012; Ouellet, Santiago, Funes, & Lupiáñez, 2010b). Although the horizontal time-space association was registered for all three temporal unit types, the measures in which it emerged differed between the unit types (see in detail below). Together with the previous studies, these new findings suggest that hours of the day, days of the week, and months of the year rely upon a relatively uniform horizontal mapping. However, the strength and the consistency of this mapping may be influenced by other spatial arrangements from calendars, grids, and hardware devices, for example, watches, phones, computer keyboards, etc. (e.g., Leone et al., 2018). We will now discuss our findings in detail and separately for each type of temporal words, providing potential explanations for differences in the strength of their associations with horizontal space.

For months of the year, the hypothesized spatial bias was observed in the initial line-intersection coordinates: We found a rightward shift in participants' responses for right-biasing month units compared to left-biasing month units. This finding demonstrates an automatic shift of visual attention caused by horizontal time associations in line with previous reports (e.g., Gevers et al., 2003; but see Price & Mentzoni, 2008). However, since we found this effect only at initial line intersections but not in the final perceptual judgments, we hypothesize that horizontal associations for months may be relatively weak and obscured by other, more dominant arrangements, such as circular clock dials (Laeng & Hofseth, 2019; Leone et al., 2018; Seymour, 1980; Zorzi et al., 2006). Indeed, some previous studies also failed to find a stable horizontal mapping of month units (e.g., Price & Mentzoni, 2008; Zorzi et al., 2006), supporting this suggestion.

For days of the week, we detected a difference in movement trajectories between left- and right-biasing stimuli: At the beginning of the cursor movements, participants exhibited the expected response bias when processing both left- and right-biasing stimuli. This finding is consistent with previous research (e.g., Gevers et al., 2004; Leone et al., 2018; Malyshevskaya et al., 2022), suggesting an automatic horizontal spatial arrangement for days of the week starting with Monday on the left and finishing with Sunday on the right. However, as with month units, the sensorimotor effect for day units appeared during the early stages of semantic processing but not in the final bisection response coordinates (or at least not during the initial line intersection). Later in the text, we discuss possible explanations for these results.

For hours of the day, an attentional shift was observed in line-bisection coordinate responses: Participants exhibited a leftward bias after listening to left-biasing hour units as compared to right-biasing hour units. To our knowledge, this is one of the first studies showing the horizontal mapping of hour units, while previous research mostly investigated their circular clockface arrangement (e.g., Bächtold et al., 1998; Bock et al., 2003; Goolkasian & Park, 1980; Ristic et al., 2006; Vuilleumier et al., 2004). Note, however, that our hour stimuli were compositional and included, in accordance with the norms of the experimental language, both numerals and words representing parts of the day (e.g., 5 a.m.: “пять часов утра” – literally “five o’clock in the *morning”* / 4 p.m. “четыре часа вечера” – literally “four o’clock in the *evening”*). Therefore, the obtained effect for hour units might in principle also be attributed to those parts of the day (morning, evening) that are spatially arranged from left to right (e.g., Ding et al., 2015).

We also observed a reverse effect for hour units in the movement trajectories: Specifically, trajectories for left-biasing hour units extended significantly more rightward when compared to trajectories for right-biasing hour units (see Fig. 2B). This reverse effect emerged between the 30th and the 41st normalized data points, i.e., in the middle part of the movement trajectory (approximately 360–490 ms after the movement onset). Note, however, that a very similar pattern can be observed in nearly all other analyses for other time units (see Fig. 2A, B, and D), although the difference between trajectories failed to reach significance in those analyses. We hypothesize that this reverse effect could be explained by overcompensation, which participants exhibited before completing the line bisection. In other words, our participants might have overcorrected the initially more left-directed trajectories for left-biasing time units, which resulted in the later rightward shift of those trajectories. The opposite could have been the case for right-directed trajectories in trials with initially more right-biasing time units.

As mentioned above, our study contributes significantly to the field by examining the dynamic and continuous accrual of spatial biases during the processing of temporal concepts. Our findings are supported by previous results (e.g., Grasso et al., 2022), suggesting that it is possible to activate the MTL even if time is irrelevant to the main task. At the same time, both our verification questions and the task itself were intentionally time-irrelevant, thus avoiding time comparisons that might involve the MTL. This could be a potential reason for the apparent instability of horizontal MTL activation for both months of the year and days of the week. Recall that previous studies failed to register space-time association when the task did not require temporal reasoning (e.g., Maienborn et al., 2015; Ulrich & Maienborn, 2010). Considering the possibility that months of the year and days of the week are not strongly associated with the horizontal dimension, it may be especially hard to detect their MTL activation when time is task-irrelevant. However, such conclusions should be drawn with caution since one could argue that the verification questions used in our study still refer to the class of the time unit (e.g., “Did the word you just heard refer to a month of the year?”), thus they could nonetheless activate the temporal reference of the stimulus. Future studies could address these factors by systematically modulating the task content and specific requirements. Another novelty of our study is introducing a new language (Russian), previously underused in such studies, to this line of research. Expanding the experimental language base is essential for examining cross-linguistic validity of the cognitive processes at hand (Blasi et al., 2022).

Together, our findings support the notion of a horizontal mapping of temporal concepts although further investigation is necessary, especially given (1) the paucity of studies directly comparing processing of different time units within the same task, and (2) the somewhat inconsistent mapping patterns observed both here and in previous studies. One of the limitations of the present study is that auditory stimuli were presented in full before the mouse movement initiation. Therefore, we may have registered only a residual tail of the putative lexical-semantic access process in movement trajectory data. To efficiently observe sensorimotor effects while processing time concepts, future research could allow for motoric responses (like mouse movement) right from the onset of auditory presentation or even earlier (see, e.g., Fischer & Hartmann, 2014). Furthermore, future research should consider using stimuli of comparable letter length, syllable length, and frequency and also control for other surface features. Another limitation of this study is that stimuli for hour units were complex and consisted of both numbers/numerals and words denoting parts of the day (a.m./p.m. – morning/evening). Therefore, it is hard to conclude whether the effects of horizontal mapping of these units appeared due to numerical information, words denoting parts of the day, or the combination of both. Thus, future studies of hour units in languages with similar syntactic structures should carefully address this issue to avoid potential confounds originating from spatial-numerical associations.

Future research focusing on the time course of sensorimotor effects might benefit from supplementing mouse tracking with other continuous methods, such as eye-tracking or neuroimaging methods (particularly time-resolved ones, such as EEG or MEG). In addition, the present findings and the techniques aimed at fine-grain scrutiny of the cognitive processing of temporal semantics can inform theories of temporal cognition in general. For example, our approach could be used in studies investigating temporal duration representation, the feeling of time, and the cognitive processing of time events. Covering multiple levels of time units is important to establish the generality of findings across scales. Moreover, the use of movement tracking during the bisection task (Allan & Gibbon, 1991; Wearden, 1991) could reveal the dynamics of temporal processing by comprehensively studying early, intermediate, and late aspects of performance.

To conclude, by employing the line bisection task, we found evidence for a horizontal time-space association for different types of time units. Notably, the effect was registered at both early (reflected in movement trajectory and initial line-intersection) and later (reflected in the final bisection response) stages of cognitive processes. Together, our findings lend unequivocal support to the general nature of the horizontal MTL and thus also support universalistic views on spatial-temporal mapping. Moreover, the present results show that spatial signatures of the MTL are an early and constitutive part of concept activation; thus, the results speak against strict disembodied/amodal representational accounts of conceptual knowledge. Overall, the present results, combined with a body of other existing evidence, support an embodied view of the processing of abstract language, including time concepts.

## Data Availability

The data reported here are accessible via the Open Science Frame work at: https://osf.io/7w9nb/?view_only=9e55f568795c4912a94ddf87ae834db1. The experiment was not preregistered.

## Footnotes

1 Note that cursor trajectory data presented in Figs. 2 and 3 are trimmed at approximately 700 pixels along y-axis coordinates as no detectable continuous coordinate differences between curves were detected in the remaining time window. Initial line intersection and bisection response coordinate data are presented in Figs. 4 and 5.
